# Researching the Co-Existence and Continuity of Standardization and Customization in Healthcare: A Response to Recent Commentaries

**DOI:** 10.15171/ijhpm.2018.07

**Published:** 2018-01-24

**Authors:** Russell Mannion, Mark Exworthy

**Affiliations:** Health Services Management Centre, University of Birmingham, Birmingham, UK.

We thank all those who responded to our article: *‘(Re) making the Procrustean Bed: Standardization and Customizations as Competing Logic in Health Care.’*^1^ Each commentary engaged critically with the main ideas we presented, offering some affirmation but also taking the discussion in new (and sometimes unexpected) theoretical and empirical directions. Each helped to shift and shape our thinking as we take forward the research agenda in this increasingly important but under researched area of contemporary health policy and management. Here, we offer a response to those commentaries and offer an agenda to shape further research.

Lena Ansmann and Holger Pfaff are supportive of many of our ideas^2^ but usefully introduce the analytical distinction in healthcare contexts between customization, personalization and individualization. In this framing, customization is conceived as those policies which serve to tailor standardized treatment and diagnosis to the psychological, social, and cultural dimensions of the patients. Personalization is viewed as the adoption of medical treatment that is aligned to the genomic and molecular profile of patients. Individualization is put forward as the generic term for the adaption of interventions to fit with a patient’s biological, psychological, social and cultural background. This logic leads Ansmann and Pfaff to the concept of ‘Individualized standardization’ - the imposition of standards, regulations or norms which “are tailored to the genes, body condition, culture, social environment, values, needs and preferences of the individual patient.” We think there is scope for research into their assertion that at the micro level, individualization may be more important than standardization, whereas on the macro level standardization is a key concern for policymakers.

Ewan Ferlie^3^ focuses on the theoretical implications of our editorial, and extends and refines these, drawing on both Foucauldian and Institutionalist perspectives. Taking a Foucauldian approach, he notes how, in many healthcare contexts, standardized risk management and clinical audit techniques have become all pervasive technologies of organizational control. He also highlights Later Foucauldian work exploring ‘technologies of the self’ which can be used to understand better contemporary personalization policies whereby individuals are seen as having the capacity to work on their own identities and make ‘positive life style choices.’ Noting that the Institutionalist Logics literature is a recent sub-stream of wider institutionalist literature, he offers a number of fruitful ways for better operationalising this approach for exploring ongoing changes at the micro-level and in particular the impact of the growing market-based logic in healthcare generated from a growing social base of informed and active users/customers.

David Greenfield, Kathy Eljiz and Kerryn Butler-Henderson^4^ highlight how processes of standardization have always been an element of health professional practice. As a result, resistance to standardization has emerged from both within and external to the healthcare professions. They lend support to our argument that as ‘competing’ logics in healthcare, standardization and customization are also long standing ‘colluding’ logics in that they both combine to hold both health professionals and patients ‘accountable’ for their expectations, thoughts and behaviour. However, they also point to a ‘fracture’ through the inconsistent and contested application and translation of standardization and customization requirements for professionals and patients. Their argument is that remedies to this situation require collaboration between professionals and patients which need to be supported by the application of systems thinking and the deployment of multiple strategies for changing values and behaviour.

Etienne Minvielle^5^ argues that efforts to customize care by tailoring it to the personal characteristics of patients and engaging patients more extensively in decisions about their own care, could result in a range of beneficial outcomes. He outlines three qualifying preconditions for developing effective customized care. First, distinct categories of patient profiles need to be used as way to explore consumer needs and in particular the clinical and genomic criteria that form the basis for personalized medicine, supplemented by additional data describing the patient’s socio-economic context. Second, the potential of IT needs to be leveraged to enable large-scale customization through facilitating the use the use of large volumes of data to help build patient categories. Third, training in customer service competencies need to foster face-to-face relationships which can contribute to the quality of customized service by enhancing aspects such as customer satisfaction, trust, loyalty, and commitment. “bedside manner.”

Catherine Needham^6^ raises the interesting question: why do these two logics always co-exist in health systems? In seeking to answer her own question, she presents possible explanations as well as exploring a number of underlying epistemological and ontological issues. In essence, her argument is that, rather than viewing the ongoing tensions between customization and standardization as indicative of an evolution from one era (standardized modernism) to the next (customized postmodernism), it may be more fruitful to seek a more pragmatic functionalist explanation: namely, that some activities which health systems undertake are inherently more amenable to standardised interventions, whereas others require approaches tailored to the particular needs and preferences of individual patients. We are sympathetic to this view and think there is scope for further theoretical and practical research in this area.

Mike Saks^7^ contends that we (implicitly) focused on the National Health Service in England without examining whether and why variations exist between countries, given the different socio-political backgrounds of particular societies. In particular, he questions how far our analysis of competing logics fits developing societies many of which are based on more traditional systems of healthcare. We agree with this criticism and think that a more international extension of our ideas would be useful and facilitate comparative research in this important area of health policy. Saks also admonishes us for ignoring the user perspective in our analysis of institutional logics. Again, we agree that exploring the consumer dimension is often missing from studies examining institutional logics. More sustained work exploring the implementation (and resistance to) standardization and customization policies across a range of healthcare settings would be a fruitful avenue for future research.

These commentaries offer an elaboration on our original article. As a result, we can draw conclusions which form the basis of an emerging research agenda in this standardization/customization debate in healthcare. This agenda comprises three elements. First, this debate provides an opportunity to incorporate more theoretically-informed, multi-disciplinary perspectives into contemporary, practical health policy considerations. In doing so, research is able to move beyond any single health system and indeed should be comparative, wherever possible. Second, the multiple levels at which standardization/customization is enacted offers a strength to address micro level issues at the same time as macro ones. As our article and commentaries demonstrate, the debate is not a binary one; indeed, greater clarification of terms, their meanings and interpretations is essential. Therefore, the co-existence of and reactivity between these levels is worth further inquiry. Furthermore, the implementation and impact of hybrid forms (such as mass customisation or individualized standardization) is under-researched. Hybridity is often the focus of research in related fields (such as institutional theory and sociology of professions) but should be investigated further here too. Third, the role of agency at each level is crucial and its significance is enhanced by the notions of multiple levels, multi-disciplinarity and hybridity. In particular, there is a compelling need to combine research into institutional and professional perspectives with those of patients and public, and crucially, the interaction between (apparently competing) perspectives. In summary, it was the intention of our original article to stimulate debate and further inquiry. The commentaries seem to confirm this. However, the debate in this journal (and elsewhere) needs to be extended and developed further through more in-depth empirical inquiry and crisper theoretical elaboration.

## Ethical issues

Not applicable.

## Competing interests

Authors declare that they have no competing interests.

## Authors’ contributions

Both authors contributed equally to the writing of this article.
